# “Lure fishing” strategies by *Mothocya parvostis* (Isopoda: Cymothoidae): Feeding behavior-mediated infestation on juveniles of black sea bream, *Acanthopagrus schlegelii*

**DOI:** 10.1016/j.ijppaw.2025.101057

**Published:** 2025-03-17

**Authors:** Hiroki Fujita, Kentaro Kawai, Michitaka Shimomura, Tetsuya Umino

**Affiliations:** a Seto Marine Biological Laboratory, Field Science Education and Research Center, Kyoto University, 459 Shirahama, Wakayama, 649-2211, Japan; b Graduate School of Integrated Sciences for Life, Hiroshima University, 1-4-4 Kagamiyama, Higashi-Hiroshima, Hiroshima, 739-8528, Japan

**Keywords:** *Artemia*, Cymothoid, Fish parasite, Infestation experiment, Infestation strategy, Laboratory condition, Manca, Optional intermediate host

## Abstract

Cymothoidae Leach, 1818 (Isopoda) are parasitic crustaceans that infest fish inhabiting marine, brackish, and freshwater environments. Few studies have examined the strategies Cymothoidae use to parasitize their hosts. In this study, we tested the hypothesis that *Mothocya parvostis* Bruce, 1986 (Isopoda: Cymothoidae) parasitizes its hosts by exploiting its feeding behavior. In our infestation experiments, juveniles of the black sea bream *Acanthopagrus schlegelii* (Bleeker, 1854) were infested with *M. parvostis* mancae in water tanks with and without nauplii of *Artemia* Leach (1819) (*A. schlegelii* feed). Overall, 46 of 100 *A. schlegelii* juveniles were parasitized, 36 of which were parasitized when they attempted to consume the mancae. The presence of *Artemia* resulted in a significantly lower infestation prevalence and a longer time to infestation. This may be attributed to the presence of *Artemia* diverting the attention of *A. schlegelii* juveniles and reducing their feeding on mancae. *Mothocya parvostis* exploits the feeding behavior of its host to increase its infestation success, similar to “lure fishing,” which may help maintain its high prevalence in hosts.

## Highlights

• *Mothocya parvostis* exploits host feeding behavior to parasitize fish.
• Parasitism decreased with the presence of *Artemia* nauplii.
• Infestation rate dropped and time to infection increased with *Artemia.*
• "Lure fishing" strategy aids *Mothocya parvostis* in maintaining high prevalence.

## Graphical abstract

**Figure:**
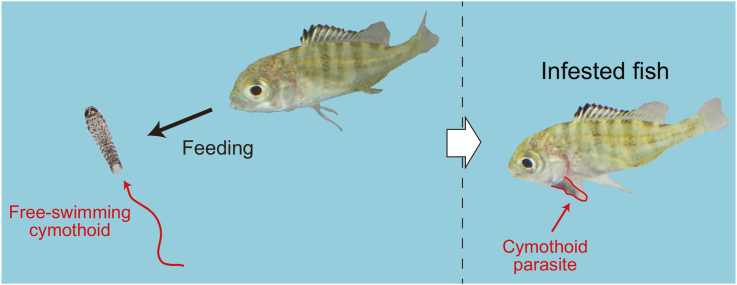

## 1 Introduction

Parasites use various strategies to enhance fitness in their relationships with their hosts. For example, the behavioral patterns of active host-invading parasites are almost perfectly adapted to maximize transmission success (Haas, 2003). Using chemicals, some parasitic worms detect and identify snails as intermediate hosts (Haas, 2003), and some parasitic isopods detect their hosts in water (Cook and Munguia, 2013; Vondriska et al., 2020). Other parasites exploit host behavior to efficiently move from intermediate to final hosts. For example, *Dicrocoelium dendriticum* (Rudolphi, 1819) Looss, 1899 (Plagiorchiida: Dicrocoeliidae) is thought to exploit the behavior of its intermediate host, the ant, to increase the efficiency of its transfer to the final sheep host (Carney, 1969).

Cymothoidae Leach, 1818 (Crustacea: Isopoda), a fish parasite, belongs to a large family containing more than 360 species in 49 genera and is distributed worldwide (Ahyong et al., 2011; Smit et al., 2014; Boyko et al., 2025). Cymothoidae are found in the branchial cavity, buccal cavity, burrowed flesh, and body surface of marine, brackish, and freshwater fishes (Smit et al., 2014). The life stages of cymothoids include manca, juveniles, males, transitional (undergoing sex change), and females (Cook and Munguia, 2015). Mancae freely swim and search for host fish to infest. They grow into juveniles and adult males on their hosts, after which the adult cymothoids change their sex from male to female. A few studies conducted on the infestation behavior of Cymothoidae have suggested that mancae encounter their hosts either by actively searching for them or through random encounters (Segal, 1987; Cook and Munguia, 2013). However, cymothoids typically release only 300–600 mancae at a time (Brusca, 1978, 1981), and the idea that random host encounters serve as the primary mechanism for finding hosts is doubtful because the movement of small animals in a viscous medium is costly and highly ineffective (Schmidt-Nielsen, 1984). In addition, the risk of parasites being excessively dispersed endangers mating success (Mikheev et al., 2015).

*Mothocya parvosti*s Bruce, 1986 (Isopoda: Cymothoidae) parasitizes the branchial cavity of fish (Bruce, 1986), infesting the Japanese halfbeak, *Hyporhamphus sajori* (Temminck and Schlegel, 1846) as a definitive final host, as well as juvenile fish such as the black sea bream, *Acanthopagrus schlegelii* (Bleeker, 1854), as optional intermediate hosts (Fujita et al., 2020, 2023b). Despite the high population density of *A. schlegelii* juveniles in Hiroshima Bay, with a maximum of 35.83 individuals per 100 m^2^ (Kawai et al., 2019), the prevalence of *M. parvostis* in these juveniles has reached 79.5 % (Fujita et al., 2020). Therefore, *M. parvostis* may have an efficient strategy to parasitize *A. schlegelii* juveniles. The parasitic strategy of *M. parvostis* may be related to the feeding behavior of its host, as the infestation site is the branchial cavity close to the mouth.

The purpose of this study was to determine the infestation strategy of *M. parvostis* on its host *A. schlegelii* juveniles. In this study, we test the hypothesis that *M. parvostis* parasitizes its hosts by exploiting feeding behavior of hosts. If *A. schlegelii* are effectively parasitized by feeding on mancae, it is expected that the prevalence of parasitism will decrease if there are many other preys. We conducted infestation experiments on *A. schlegelii* juveniles with and without the presence of prey (*Artemia* sp.), and compared the prevalence and time taken to complete the infestation.

## 2 Materials and methods

### 2.1 Collection of experimental organisms

The non-infested *A. schlegelii* juveniles (standard length <2 cm) used in the experiment were collected in June 2022 and June 2023 using a hand net in the estuary of the Kamo River in Takehara City, Hiroshima Prefecture, Japan. Infested fish can be easily distinguished by sight because the pleotelson of *M. parvostis* protrudes from the gill (Saito and Yoneji, 2000). They were reared in a tank (80 cm in diameter and 50 cm in height) filled with 150 L of filtered seawater and connected to an external filtration system, NJC-584 (NISSO, Tokyo, Japan), or running seawater. During rearing, fish were fed an artificial diet daily. Cymothoid mancae were collected from the west coast of Nomi Island, Etajima City, Hiroshima Prefecture, in June 2022 and June 2023 by light trapping according to Fujita et al. (2023c). The *M. parvostis* mancae were separated from juveniles by body size, based on Fujita et al. (2023c), and reared in seawater for further experiments.

### 2.2 Infestation experiments

**Fig. 1:**
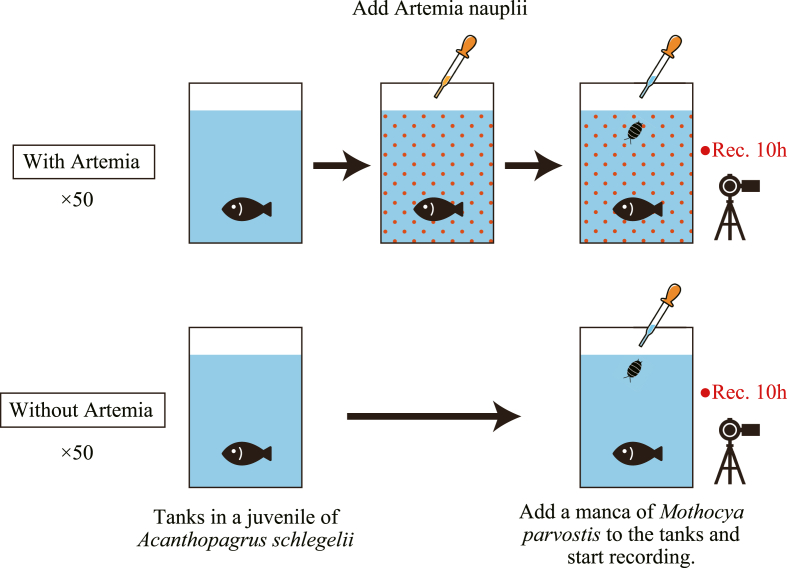
Overview of the experimental design.

Juvenile *A. schlegelii* infestation experiments were conducted using *M. parvostis*. If the parasite exploits host-feeding behavior, an increase in the number of prey animals is expected to change the infestation success rate. One experiment consisted of the following steps. A glass water tank (10 × 6.5 × 14.2 cm) was filled with 700 mL of filtered seawater (temperature, 23 °C; salinity, 2.8), and a non-infested juvenile of *A. schlegelii* was placed in the water tank. Next, one *M. parvostis* manca was dropped into each seawater tank using a Komagome pipette. The tank was video-recorded from the front using a GoPro HERO10 video camera (GoPro, CA, USA) for 10 h after the addition of the manca. After 10 h, *A. schlegelii* juveniles were removed from the tank and checked for infestation. If the fish were infested, the time of infestation was recorded from the video. Adobe Premiere Pro (Adobe, CA, USA) video editing software was used to join the videos and measure the time of infestation. The presence or absence of parasites was determined by observing the gills of the fish at each time point in the video. The moment of infestation can be determined from the fish struggling (swimming irregular with wide-open gills and mouth) in the video. We conducted the experiment 50 times, with and without prey (a total of 100 times, using 100 fish and 100 mancae), to determine whether the infestation success rate differed (Fig. 1). Nauplii of *Artemia* sp., commonly used as feed in *A. schlegelii* seedling production (Keitoku et al., 1985), were used as prey in this study to divert the attention of *A. schlegelii* juveniles. Approximately 3000–juvenile *A. schlegelii* would not be able to eat during the experiment period–hatched *Artemia* were added to the tanks with *Artemia* nauplii. The experiment was conducted in 10 tanks. The dates of fish collection, manca collection, and experiment ID for each experiment are shown in Supplementary Table S1.

R software (R Foundation for Statistical Computing, Vienna, Austria) (Ihaka and Gentleman, 1996) and the EZR package (Easy R version 2.4-0) (Kanda, 2013) were used for statistical analyses. The effect of the presence or absence of *Artemia* on the infestation success rate was examined using the χ^2^ test. The time taken to reach infestation was compared using the Mann–Whitney *U* test.

### 2.3 Molecular identification of mancae

The mancae used in the experiment were collected as plankton and could not be fully identified by morphology because taxonomic traits of Cymothoidae are underdeveloped in growth stages other than the female stage (Fujita et al., 2021; Fujita, 2023, Fujita et al., 2023d). Therefore, using the cytochrome *c* oxidase subunit I (COI) sequence, we confirmed that the mancae were *M. parvostis*. The number of individuals analyzed was set to an effective sample size of 24, calculated with an *α*-error of 0.05, 100 individuals, a confidence level of 0.95, and a population proportion of 0.98 [percentage of *M. parvostis* among mancae collected in June in Fujita et al. (2023c)].

DNA extraction and PCR amplification were performed as described by Fujita et al. (2023a). PCR products were sent to Eurofins Genomics (Tokyo, Japan) sequencing services and sequenced using the dye terminator method. The sequences have been deposited in GenBank (accession numbers: PV297929–PV297952). The Basic Local Alignment Search Tool (BLAST) was run on each sequence in the NCBI GenBank. We established confidence values for identification as ≥ 99 % similarity and E-value = 0.0.

## 3 Results

### 3.1 Infestation experiments

**Fig. 2:**
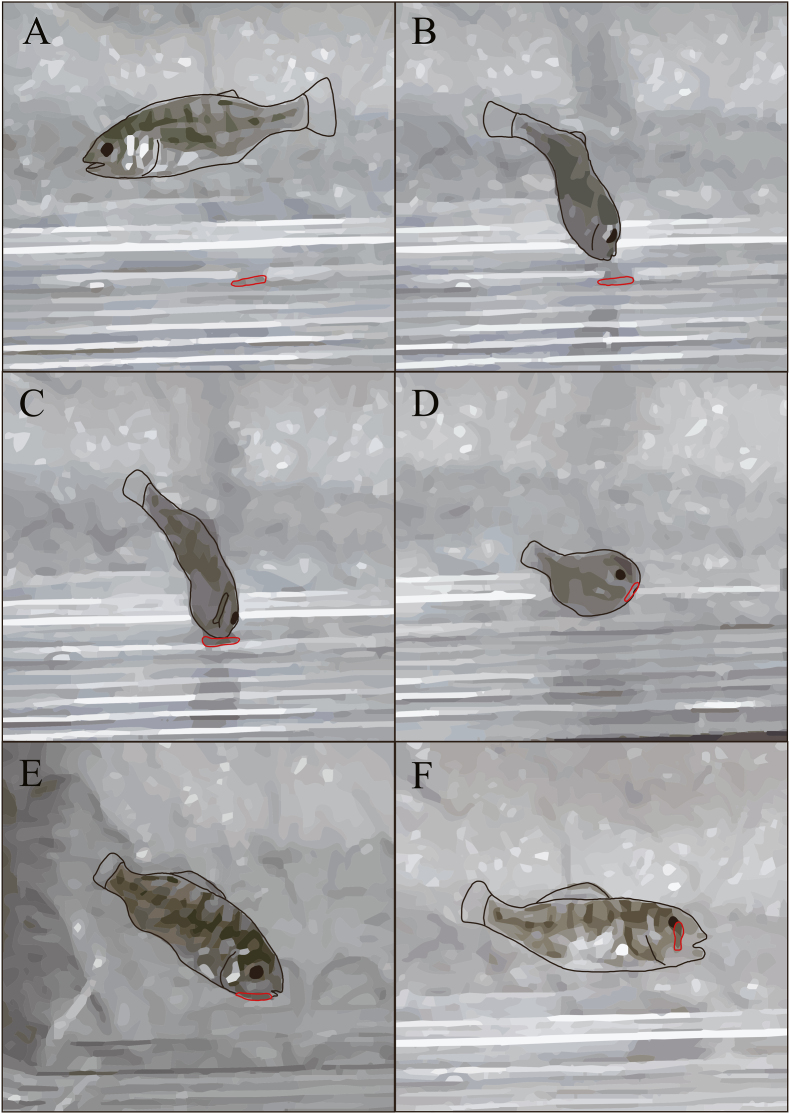
Illustration created from the video showing the moment that juveniles of *Acanthopagrus schlegelii* (Bleeker, 1854) were parasitized by *Mothocya parvostis* Bruce, 1986 manca. A–F in time order. D: struggling *A. schlegelii* juvenile. Black lines show *A. schlegelii* juvenile and red lines show *M. parvostis* manca.

**Fig. 3:**
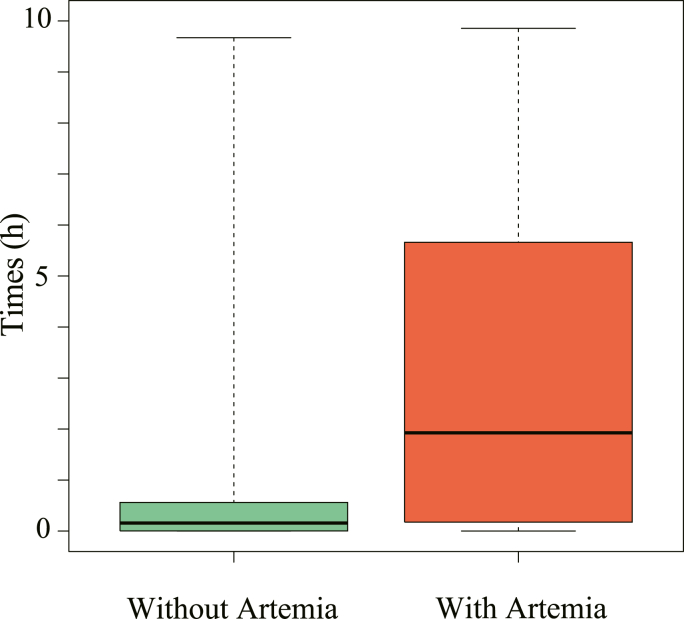
Comparison of the time required for *Mothocya parvostis* Bruce, 1986 mancae to parasitize to *Acanthopagrus schlegelii* (Bleeker, 1854) juveniles with and without *Artemia* nauplii.

Of the 100 juveniles of *A. schlegelii* used in the experiment, 46 (30 without *Artemia*, 16 with *Artemia*) were parasitized by *M. parvostis* during 10 h of the experiment (Fig. 2). In addition, juveniles of *A. schlegelii* actively fed on *Artemia* in tanks to which *Artemia* was added. The χ^2^ test showed that the presence or absence of *Artemia* affected the parasitic success rate (*p* = 0.00091), with a prevalence of 60 % without *Artemia* and 32 % with *Artemia*. The mean time from manca addition to infestation was 1:02:12 ± 2:09:00 (h: min: s) (±SD) in the absence of *Artemia* (*n* = 30) and 3:11:50 ± 3:22:07 in the presence of *Artemia* (*n* = 16), with significant differences between the presence and absence of *Artemia* (Mann–Whitney *U* test, *p* = 0.026) (Fig. 3).

**Fig. 4:**
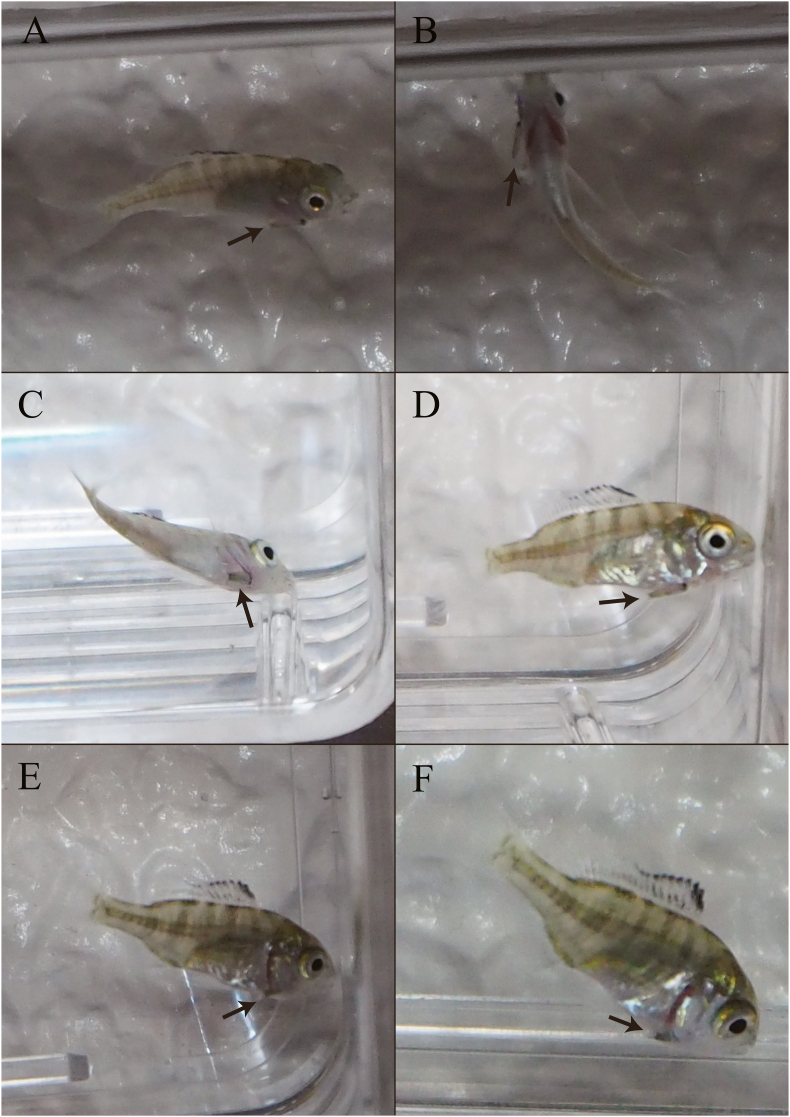
Images showing *Mothocya parvostis* Bruce, 1986 manca migrating from the body surface to the branchial cavity of *Acanthopagrus schlegelii* (Bleeker, 1854) juveniles. A–F in time order. Arrows indicate the manca.

Video observations confirmed infestation. Of the 46 *A. schlegelii* juveniles confirmed to be infested, 36 were infested with mancae while attempting to feed on them (Fig. 2, Video 1). In some cases, infestation was due to juvenile *A. schlegelii* chasing the swimming manca (*n* = 16), whereas in other cases, *A. schlegelii* found the manca motionless at the bottom and tried to feed on it (*n* = 20) (Fig. 2). The remaining 10 *A. schlegelii* juveniles were parasitized by accidental swimming contact–it is occurring when *A. schlegelii* juvenile was not facing the manca. *Mothocya parvostis* first attached to the body surface of *A. schlegelii,* and then migrated to the gills and parasitized the branchial cavity (Fig. 4). When the fish were parasitized during feeding, the migration time to the branchial cavity was within 10 min for 58.3 % of the 36 individuals, between 10 and 20 min for 5.6 %, and >30 min for 36.1 % (including individuals who did not complete migration within the experimental time). In the case of infestation by accidental contact, the migration time to the branchial cavity was ≤10 min for 10 % of the 10 individuals and >30 min for 90 %. One of the 100 *M. parvostis* mancae used in the infestation experiment was swallowed by *A. schlegelii* juveniles during feeding; however, no migration into the branchial cavity was observed.

### 3.2 Molecular identification

The 24 COI sequences were >99 % identical to those of *M. parvostis* according to BLAST research (Supplementary Table S2). Thus, all mancae used in the infestation experiments were regarded as *M. parvostis*.

## 4 Discussion

*Acanthopagrus schlegelii* is an omnivore, and its juvenile prey include small crustaceans, including Sphaeromatidea Latreille, 1825, Amphipoda, Tanaidacea, and Copepoda (Shigeta and Tomiyama, 2021). Sphaeromatidea are of the same order (Isopoda) as *M. parvostis*, and juveniles of *A. schlegelii* may consider *M. parvostis* mancae and juveniles, which are similar in appearance to their prey. In the present study, most *A. schlegelii* juveniles were parasitized when they voluntarily approached mancae. In addition, the infestation success rate decreased in the tanks in which *Artemia* nauplii was added. This may be because *A. schlegelii* juveniles fed on *Artemia* in tanks to which they were added, reducing the frequency of their feeding behavior toward *M. parvostis*. Only 10 of 46 individuals were parasitized by accidental contact. The experimental tanks were small (700 mL); therefore, the frequency of accidental contact might be lower in the natural environment. *Mothocya parvostis* is thought to parasitize *A. schlegelii* juveniles by exploiting the feeding behavior of hosts, thereby increasing the parasitic efficiency of *M. parvosti*s.

The probability of *A. schlegelii* juveniles feeding on mancae will decrease as the number of other prey species increases. Therefore, if the parasitism of *M. parvostis* is dependent on the feeding behavior of *A. schlegelii* juveniles, the infestation success rate will decrease as the number of prey increases. The results of this study were as hypothesized. However, if the infestation success rate decreases in proportion to the density of the prey, the infestation success rate should be 1 in 3000, but the infestation success rate with *Artemia* in this study was not that low. This is probably because the experimental environment was small, so accidental contact occurred in a number that could not be ignored, as well as because the mancae were larger and more noticeable than *Artemia*.

In this study, when *M. parvostis* mancae parasitized juveniles of *A. schlegelii*, they first parasitized the body surface of the fish and then migrated to the branchial cavity. The time taken to reach the branchial cavity tended to be shorter when the host was parasitized through its feeding behavior, compared to accidental contact. This strategy may be advantageous for rapid migration to the branchial cavity, as the parasites attach to the mouth area of *A. schlegelii* juveniles during feeding, thus shortening the migration distance.

Segal (1987) observed the parasitic behavior of aegathoid juveniles of *Nerocila acuminata* Schiödte and Meinert, 1881 (Isopoda: Cymothoidae) against their hosts in a laboratory setting. They reported that juveniles approached their hosts from behind or ambushed them from underneath, with their dorsal surfaces facing downward. They parasitized the fish by hooking their pereopods onto the body surface as the fish passed nearby. Aegathoid juveniles have also been reported to be predated by fish; however, the parasites are thought to be killed by feeding. Therefore, parasitic behavior through fish feeding was not observed. The current study is the first to show that Cymothoidae parasitize fish by exploiting their feeding behavior. The buccal cymothoid *Cymothoa excisa* Perty, 1833, increases motility in the presence of fish-derived chemicals, thereby facilitating infestation (Cook and Munguia, 2013). In the experiments for this study, the behavior of mancae chasing fish was not observed. Although chemotaxis and other factors underlying *M. parvostis* infestation remain unknown, these other strategies, together with feeding behavior exploitation, are likely to increase the parasitic success rate.

*Hyporhamphus sajori*, the definitive final host of *M. parvostis*, swims near the sea surface and feeds on plankton, such as copepods and decapods, but not fish juveniles (Kuniyuki and Koide, 1962; Yao et al., 2002). Because *M. parvostis* mancae and juveniles are collected near the surface where *H. sajori* swims (Fujita et al., 2023c), and infestation occurred both when the manca was swimming and when it was at the bottom in this study; therefore, it is highly likely that *H. sajori* engages in predatory behavior against these mancae and juveniles. Experiments were conducted on juveniles of *A. schlegelii*, an optional intermediate host in the present study; however, it is possible that *H. sajori*, the final host, is also parasitized via feeding behavior. Fujita et al. (2020) speculated that *M. parvostis* juveniles detach from juveniles of *A. schlegelii* after parasitizing them for a certain period, and the detached juveniles may seek to parasitize *H. sajori*. If this hypothesis is correct, the feeding behavior of *M. parvostis* may also be advantageous for the infestation of *H. sajori* after detachment from juveniles of *A. schlegelii*.

*Mothocya parvostis* is known to inhibit the growth of *A. schlegelii* juveniles (Fujita et al., 2020) and has a negative impact on the body weight of *H. sajori*, the definitive final host, in waters heavily influenced by human activity (Kawanishi et al., 2016). The effects of *M. parvostis* on these fish resources are expected to increase with its increasing prevalence. The maximum annual prevalence of *M. parvostis* in *A. schlegelii* juveniles fluctuates from 23.8 % to 79.5 % (Fujita et al., 2020). Because the infestation success rate was lower in environments with prey in this study, it is possible that one of the factors contributing to the year-to-year variation in prevalence is the amount of prey. A decrease in prey species may increase the prevalence of *M. parvostis* in fish, which may have a greater impact on the effects of infestation.

## 5 Conclusion

In the water tank experiment, we showed that *M. parvostis* exploits the feeding behavior of *A. schlegelii* juveniles to promote infestation, similar to “lure fishing.” *Mothocya parvostis* may enhance its fitness by utilizing optional intermediate hosts (Fujita et al., 2020, 2023b). These multiple infestation strategies may be responsible for the high prevalence of *M. parvostis* on *H. sajori* (approximately 50 %) (Kawanishi et al., 2016; Fujita et al., 2020), which is a polyphagous fish. This study was conducted under laboratory conditions; therefore, further verification is necessary to determine whether this parasitic strategy is applicable to natural environments.

## CRediT authorship contribution statement

**Hiroki Fujita:** Writing – original draft, Visualization, Methodology, Investigation, Formal analysis, Data curation, Conceptualization. **Kentaro Kawai:** Writing – review & editing, Methodology. **Michitaka Shimomura:** Writing – review & editing, Supervision. **Tetsuya Umino:** Writing – review & editing, Supervision.

## Data availability

The molecular data were deposited in GenBank (accession numbers: PV297929–PV297952). Raw data is containing in Supplementary Table S1.

## Ethical approval

The authors confirm that the ethical policies of Japan, the institution, and the journal were followed.

## Funding

This work was partially supported by grants-in-aid from the Japan Society for the Promotion of Science (KAKENHI No. 23KJ1170) and the Japan Science and Technology Agency SPRING (Grant No. JPMJSP2132).

## Declaration of competing interest

The authors declare that they have no conflict of interest.

## Appendix A Supplementary data

Multimedia component 2 Multimedia component 2

Multimedia component 3 Video of an infestation experiment with juvenile *Acanthopagrus schlegelii* (Bleeker, 1854) and *Mothocya parvostis* Bruce (1986) manca, without *Artemia* nauplii. Multimedia component 3
